# Current Status and Future Directions of Bone Trauma Surgery

**DOI:** 10.3390/medicina60081297

**Published:** 2024-08-11

**Authors:** Ivo Dumic-Cule

**Affiliations:** 1 University North, 104 Brigade 3, 42000 Varazdin, Croatia; ivodc1@gmail.com; Tel.: +385-98-1655-686; 2 Polyclinic Da Vinci, Petrovaradinska 110, 10000 Zagreb, Croatia

The ever-evolving field of bone trauma surgery and bone regeneration is characterized by continuous transformation due to advancements in medical technology, enhancements in surgical techniques, and a deeper understanding of biological interactions underlying the processes of bone healing and regeneration. Therefore, this Special Issue of *Medicina*, “Current Status and Future Directions of Bone Trauma Surgery”, is dedicated to presenting the latest knowledge and exploring future directions in bone trauma surgery. It comprises twelve articles focusing on all relevant aspects of bone trauma surgery and bone regeneration, including upgrades to the current techniques used for the management of bone trauma. Additionally, this Special Issue aimed to provide comprehensive reviews that can be useful and applicable to everyday traumatology practice.

Bone trauma surgery has made remarkable strides in recent decades. The integration of minimally invasive techniques, the development of sophisticated fixation devices, and the use of biologics for bone healing have revolutionized the field [1]. Currently, surgeons have a plethora of tools at their disposal, enabling them to tackle complex fractures with greater precision, resulting in improved outcomes. In this Special Issue, Mao and collaborators described less-invasive surgical techniques that yielded reduced rates of postoperative complications and faster recovery periods. In patients with distal-third humeral fractures resulting from arm wrestling, single plating has demonstrated a union rate and elbow range of motion comparable to that achieved using double plating. Moreover, significant advantages were described, including fewer complications, reduced surgical time, and lower blood loss. These benefits contribute to improved early functional outcomes, making single plating a more favorable option when treating these specific fractures [2].

Recent studies have underscored the importance of early intervention and individualized treatment plans, thereby ensuring functional recovery and, subsequently, increased quality of life for patients. Lee, S.J. and collaborators suggested that prehospital cervical and spinal immobilization should be more selectively applied to certain head and neck injury groups. This selective approach is particularly important for individuals older than 65, those with impaired consciousness (GCS 8), individuals suffering from severe traumatic injuries (ISS 16 or RTS 7), and patients experiencing shock. They emphasized a retrospective study design as well as its limitations and potential biases [3].

Although enhanced imaging technologies, such as 3D CT scans and MRI, play a crucial role in preoperative planning and intraoperative navigation, Tullio P.O.D. et al. demonstrated that there are certain conditions in which CT scans are no more useful than conventional X-rays. CT scans did not enhance the surgeons’ primary interpretation of the Hertel prognostic criteria, nor did they provide additional value compared to radiographic examinations [4].

Emerging innovations in bone trauma surgery are also promising in terms of enhancing surgeon performance. The use of augmented reality (AR) in surgery is an exciting frontier, providing surgeons with augmented visualization and enhanced precision during procedures [5]. Over the past decade, the introduction of both high- and low-fidelity virtual reality systems for bone trauma procedures has resulted in significant benefits. These systems have enhanced procedural teaching and learning, improved preoperative planning, increased intraoperative precision and efficiency, and contributed to better postoperative outcomes. Despite these advancements, there is a need for further technical developments that meet industry benchmarks and metrics. Additionally, more standardized and rigorous clinical validation is required to fully realize the potential of these virtual reality systems in bone trauma procedures.

Percutaneous vertebroplasty is a minimally invasive technique employed to treat vertebral body compression fractures, offering significant relief and functional improvement. The most common complication is cement leakage, which can infiltrate the epidural, intradiscal, foraminal, paravertebral regions, and even the venous system. While many complications are asymptomatic, they pose the potential for severe consequences. A review published in this Special Issue is based on single-center experiences, comprised of various complications associated with vertebroplasty, and aimed to improve our understanding of the pathology and mitigate objective risks [6].

Despite the variability in terminology (hypertrophic arthritis, degenerative arthritis, arthritis deformans, etc.), our understanding of osteoarthritis has advanced due to extensive in vitro and in vivo research, elucidating the disease’s pathophysiology and pathology at both histological and cellular levels. Nonetheless, the precise cause of osteoarthritis remains unknown. Therefore, a review written by Dudaric et al. consolidates the recent findings on the biological processes that occur in bone tissue during osteoarthritis, aiming to provide insights beneficial for clinical practice. Emphasizing the importance of selecting appropriate radiological techniques is crucial for the early diagnosis and effective management of this prevalent and debilitating chronic condition [7].

In summary, this Special Issue is dedicated to advancements in bone trauma surgery and bone regeneration that are transforming patient care and outcomes. Through comprehensive reviews and clinical studies, contributors have suggested modifications of surgical techniques, radiologic methods, and innovative technologies that enable better diagnostics, enhance healing, and reduce complications.
